# The genome sequence of the lesser black-backed gull, *Larus fuscus *Linnaeus, 1758

**DOI:** 10.12688/wellcomeopenres.22766.2

**Published:** 2024-10-15

**Authors:** Rosa Lopez Colom, Michelle F. O’Brien, Natural History Museum Genome Acquisition Lab, Darwin Tree of Life Barcoding collective, Wellcome Sanger Institute Tree of Life Management, Samples and Laboratory team, Wellcome Sanger Institute Scientific Operations: Sequencing Operations, Wellcome Sanger Institute Tree of Life Core Informatics team, Tree of Life Core Informatics collective, Darwin Tree of Life Consortium

**Affiliations:** 1 Wildfowl & Wetlands Trust, Slimbridge, England, UK

**Keywords:** Larus fuscus, lesser black-backed gull, genome sequence, chromosomal, Charadriiformes

## Abstract

We present a genome assembly from an individual female *Larus fuscus* (the lesser black-backed gull; Chordata; Aves; Charadriiformes; Laridae). The genome sequence has a total length of 1,324.30 megabases. Most of the assembly (90.38%) is scaffolded into 32 chromosomal pseudomolecules, including the Z sex chromosome. The mitochondrial genome has also been assembled and is 16.75 kilobases in length.

## Species taxonomy

Eukaryota; Opisthokonta; Metazoa; Eumetazoa; Bilateria; Deuterostomia; Chordata; Craniata; Vertebrata; Gnathostomata; Teleostomi; Euteleostomi; Sarcopterygii; Dipnotetrapodomorpha; Tetrapoda; Amniota; Sauropsida; Sauria; Archelosauria; Archosauria; Dinosauria; Saurischia; Theropoda; Coelurosauria; Aves; Neognathae; Charadriiformes; Laridae; *Larus*; *Larus fuscus* Linnaeus, 1758 (NCBI:txid8915).

## Background

The lesser black-backed gull (LBBG), *Larus fuscus*, resembles the herring gull ( *Larus argentatus*) except is slightly smaller and slimmer. It reaches a length of 52 to 67 cm, has yellow legs and a yellow bill with a red mark, and a darker slate-coloured back that extends across the wings, which end in even darker tips often marked with a white primary spot. The average weight ranges from 650 g to 1000 g. These gulls typically have a lifespan of 10 to 15 years ( Holden *et al.*, 2014; Hume, 2014).

As with many *Laridae* species, there are differences between their summer and winter moults. In winter, they lose their brightness; their limbs and bill take on a duller yellow pigment, their heads transition from homogenous white to densely streaked grey, and their wings also show a duller hue of grey ( Hume, 2014).

There are several recognised subspecies of the LBBG, each with slight variations in plumage and size. These subspecies include *Larus fuscus fuscus*, *Larus fuscus graellsii*, and *Larus fuscus intermedius*, among others ( Collinson *et al.*, 2008). These different subspecies can be found from Iceland to western and northern Siberia throughout the year. However, during autumn and winter, breeding populations disperse across Europe, with many migrating south along the Mediterranean coast and into northern Africa. Breeding grounds for lesser black-backed gulls commonly include lowland coastal areas, but they can also be found nesting on the rooftops of buildings in semi-urban inland regions ( Hume, 2014).

The diversity within the species provides an interesting study in evolutionary adaptation and variation. The LBBGs are known to hybridise with other gull species ( Liebers *et al.*, 2004). Genomic analysis can help clarify the extent and impact of hybridisation and understand the genetic exchange between species. This is important for both species identification and conservation management.

Under the IUCN Red List, LBBGs are considered a species of 'Least Concern'; however, their conservation status in the UK is amber due to significant population decline ( British Trust for Ornithology, 2015). Threats contributing to the decline of this species include habitat loss, pollution, a reduced food supply, fewer available nesting sites inland, and illegal killing ( OSPAR Assessment Portal, 2020).

The genome of the lesser black-backed gull, *Larus fuscus*, was sequenced as part of the Darwin Tree of Life Project, a collaborative effort to sequence all named eukaryotic species in the Atlantic Archipelago of Britain and Ireland.

## Genome sequence report

**Figure 1.**
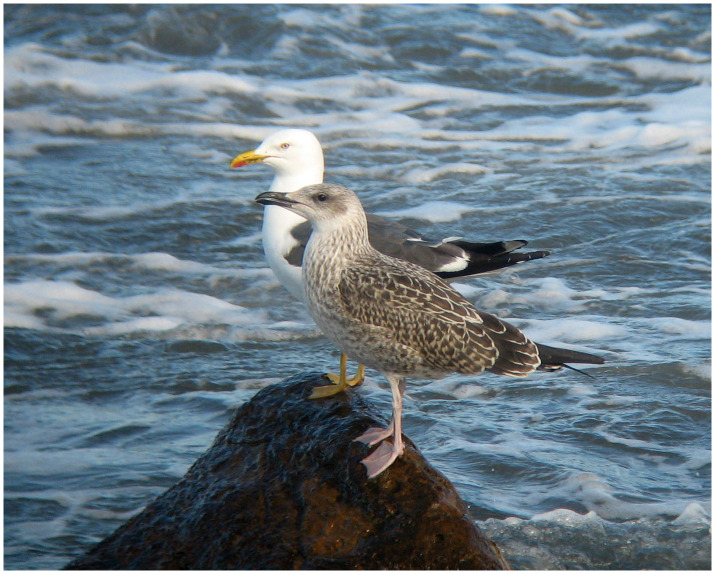
Photograph of Lesser Black-backed Gull *Larus fuscus*.

The genome of an adult female *Larus fuscus* ( Figure 1) was sequenced using Pacific Biosciences single-molecule HiFi long reads, generating a total of 57.64 Gb (gigabases) from 7.50 million reads, providing an estimated 30-fold coverage based on the initial GenomeScope genome size estimate. Primary assembly contigs were scaffolded with chromosome conformation Hi-C data, which produced 75.18 Gb from 497.87 million reads, yielding an approximate coverage of 57-fold. Specimen and sequencing details are summarised in Table 1.

**Table 1.** Specimen and sequencing data for *Larus fuscus*.

Project information			
**Study title**	Larus fuscus (lesser black-backed gull)		
**Umbrella BioProject**	PRJEB71570		
**Species**	*Larus fuscus*		
**BioSample**	SAMEA112468039		
**NCBI taxonomy ID**	8915		
Specimen information			
**Technology**	**ToLID**	**BioSample ** **accession**	**Organism ** **part**
**PacBio long read sequencing**	bLarFus1	SAMEA112468094	muscle
**Hi-C sequencing**	bLarFus1	SAMEA112468094	muscle
**RNA sequencing**	bLarFus1	SAMEA112468096	muscle
Sequencing information			
**Platform**	**Run accession**	**Read count**	**Base ** **count (Gb)**
**Hi-C Illumina NovaSeq 6000**	ERR12512742	4.98e+08	75.18
**PacBio Sequel IIe**	ERR12408797	3.14e+06	21.77
**PacBio Sequel IIe**	ERR12408796	1.88e+06	17.73
**PacBio Sequel IIe**	ERR12736858	2.48e+06	18.14
**RNA Illumina NovaSeq 6000**	ERR12512743	5.12e+07	7.73

**Figure 2.**
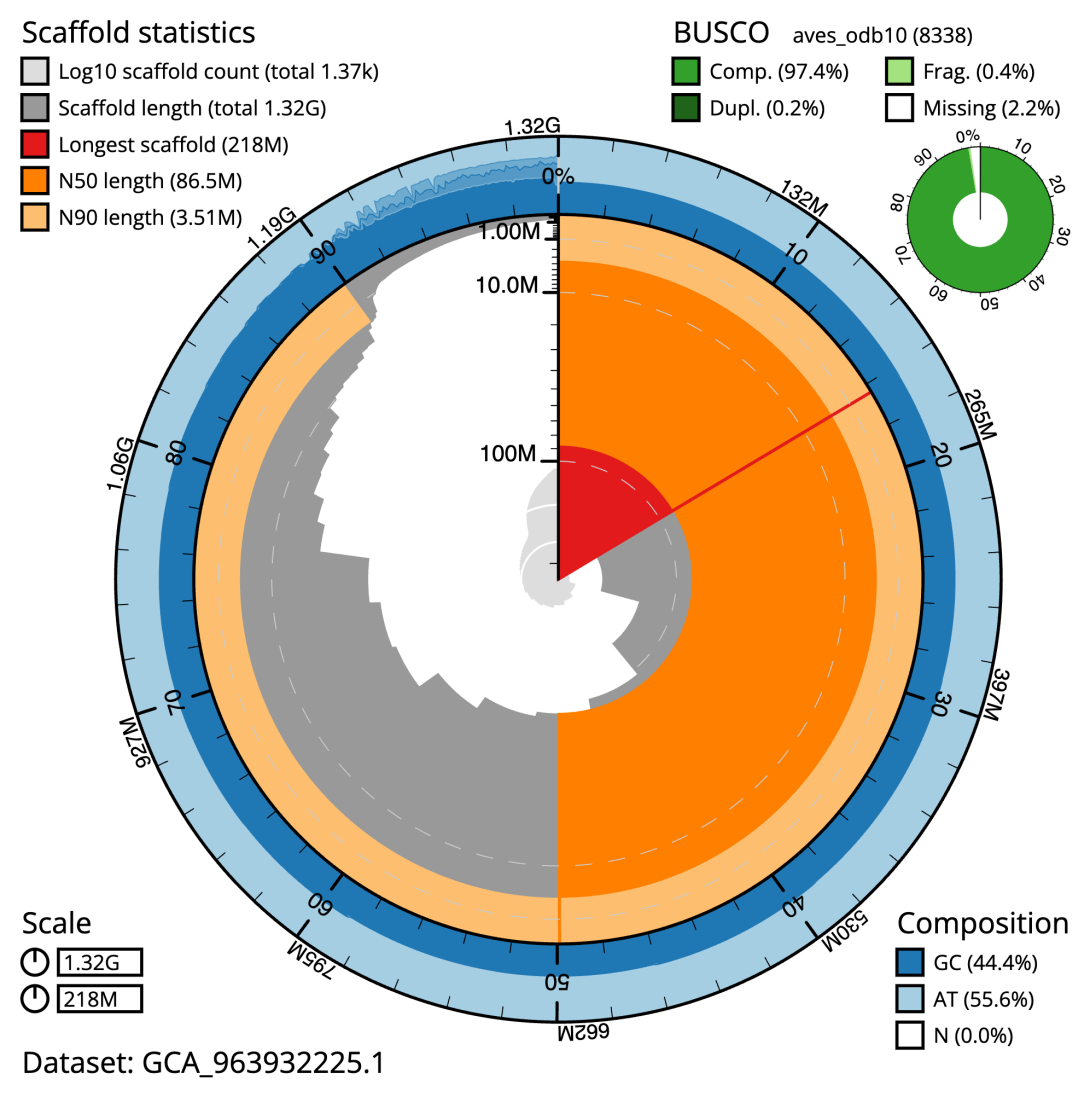
Genome assembly of *Larus fuscus*, bLarFus1.1: metrics. The BlobToolKit snail plot shows N50 metrics and BUSCO gene completeness. The main plot is divided into 1,000 size-ordered bins around the circumference with each bin representing 0.1% of the 1,324,286,540 bp assembly. The distribution of scaffold lengths is shown in dark grey with the plot radius scaled to the longest scaffold present in the assembly (218,474,787 bp, shown in red). Orange and pale-orange arcs show the N50 and N90 scaffold lengths (86,500,895 and 3,505,000 bp), respectively. The pale grey spiral shows the cumulative scaffold count on a log scale with white scale lines showing successive orders of magnitude. The blue and pale-blue area around the outside of the plot shows the distribution of GC, AT and N percentages in the same bins as the inner plot. A summary of complete, fragmented, duplicated and missing BUSCO genes in the aves_odb10 set is shown in the top right. An interactive version of this figure is available at https://blobtoolkit.genomehubs.org/view/Larus_fuscus/dataset/GCA_963932225.1/snail.

**Figure 3.**
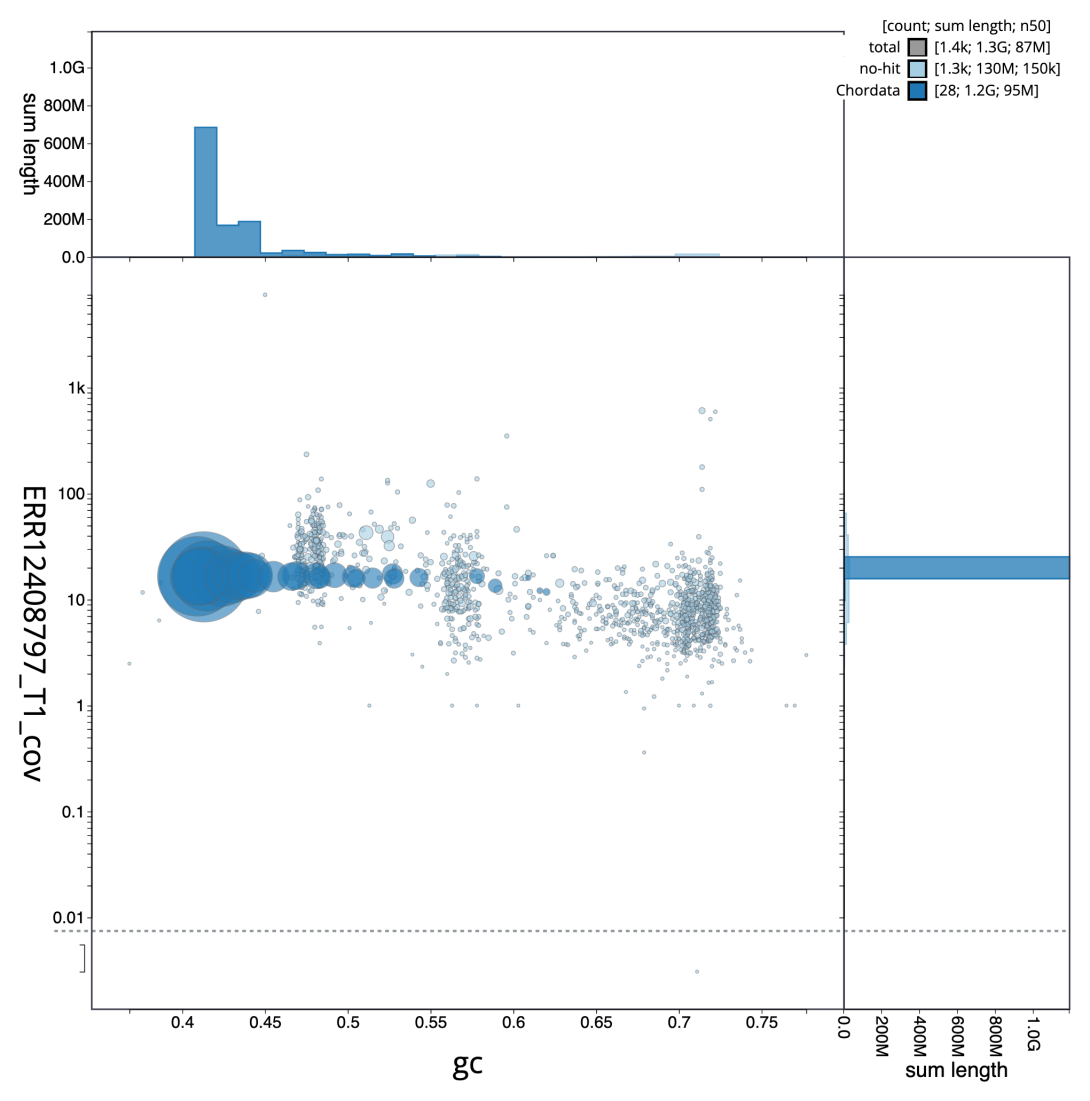
Genome assembly of *Larus fuscus*, bLarFus1.1: BlobToolKit GC-coverage plot. Sequences are coloured by phylum. Circles are sized in proportion to sequence length. Histograms show the distribution of sequence length sum along each axis. An interactive version of this figure is available at https://blobtoolkit.genomehubs.org/view/Larus_fuscus/dataset/GCA_963932225.1/blob.

**Figure 4.**
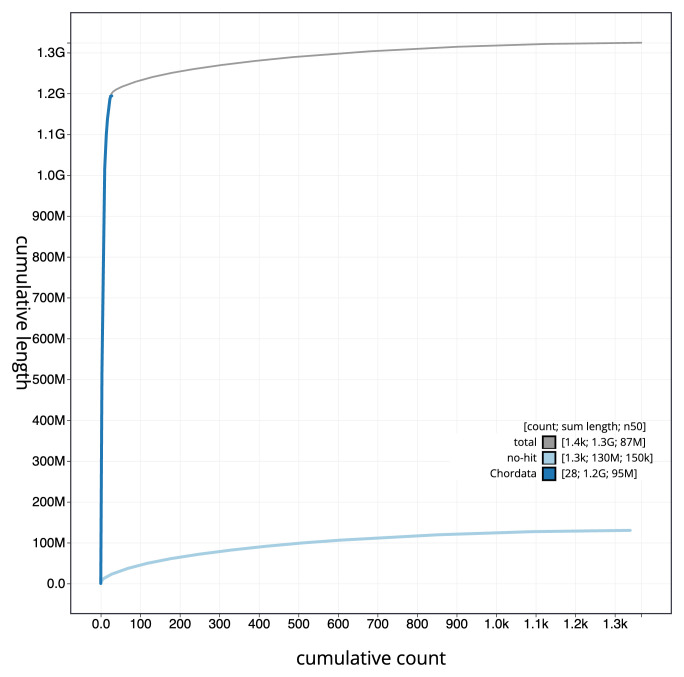
Genome assembly of *Larus fuscus* bLarFus1.1: BlobToolKit cumulative sequence plot. The grey line shows cumulative length for all sequences. Coloured lines show cumulative lengths of sequences assigned to each phylum using the buscogenes taxrule. An interactive version of this figure is available at https://blobtoolkit.genomehubs.org/view/Larus_fuscus/dataset/GCA_963932225.1/cumulative.

**Figure 5.**
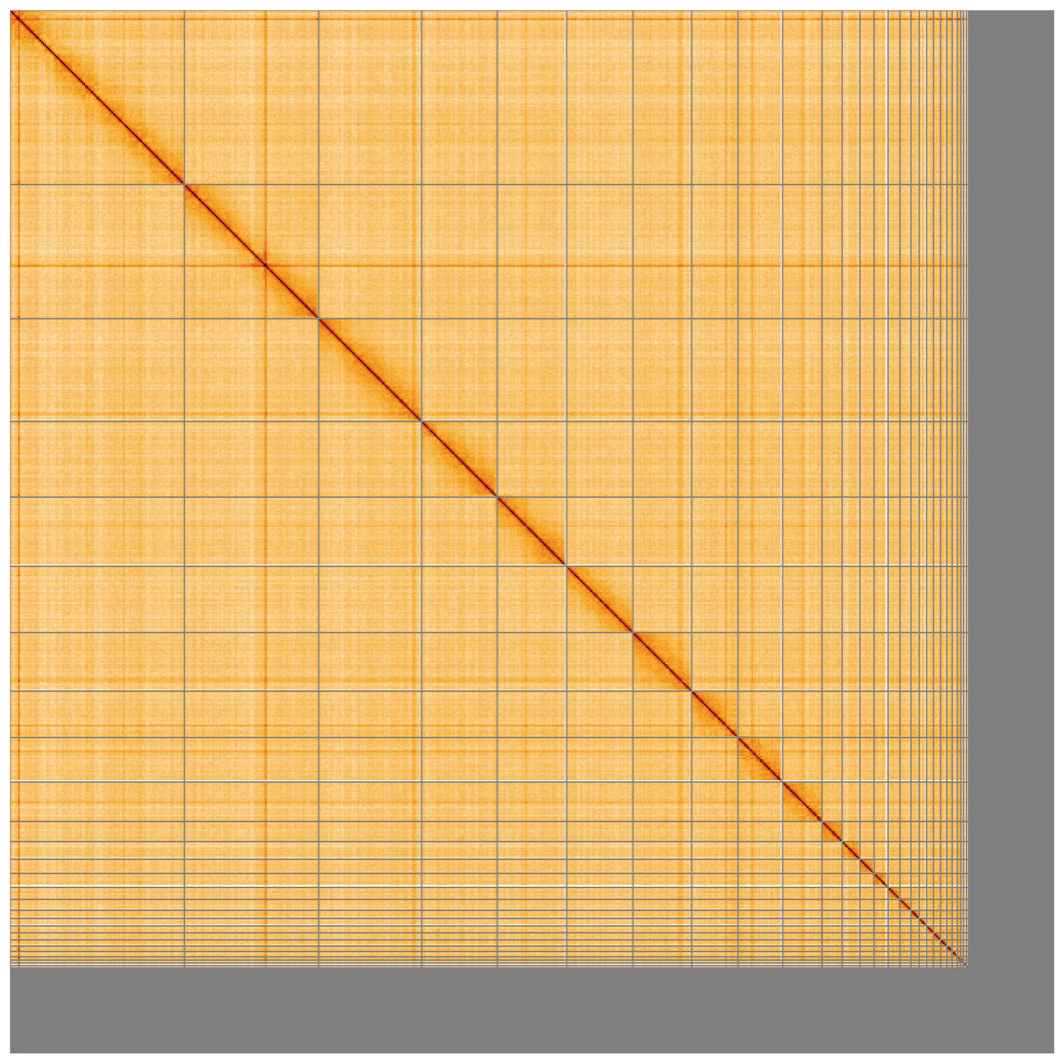
Genome assembly of *Larus fuscus* bLarFus1.1: Hi-C contact map of the bLarFus1.1 assembly, visualised using HiGlass. Chromosomes are shown in order of size from left to right and top to bottom. An interactive version of this figure may be viewed at https://genome-note-higlass.tol.sanger.ac.uk/l/?d=CQjcZyANQcysUbst203I9w.

Manual assembly curation corrected 44 missing joins or mis-joins, reducing the scaffold number by 1.94%. The final assembly has a total length of 1,324.30 Mb in 1,366 sequence scaffolds with a scaffold N50 of 86.5 Mb ( Table 2), and 537 gaps. The total count of gaps in the scaffolds is 537. The snail plot in Figure 2 provides a summary of the assembly statistics, while the distribution of assembly scaffolds on GC proportion and coverage is shown in Figure 3. The cumulative assembly plot in Figure 4 shows curves for subsets of scaffolds assigned to different phyla. Most (90.38%) of the assembly sequence was assigned to 32 chromosomal-level scaffolds, representing 31 autosomes and the Z sex chromosome. Chromosome-scale scaffolds confirmed by the Hi-C data are named in order of size ( Figure 5; Table 3). The Z chromosome was identified based on alignment with *Gallus gallus* (GCF_016699485.2). While not fully phased, the assembly deposited is of one haplotype. Contigs corresponding to the second haplotype have also been deposited. The mitochondrial genome was also assembled and can be found as a contig within the multifasta file of the genome submission.

**Table 2.** Genome assembly data for *Larus fuscus*, bLarFus1.1.

Genome assembly		
Assembly name	bLarFus1.1	
Assembly accession	GCA_963932225.1	
*Accession of alternate haplotype*	*GCA_963932325.1*	
Span (Mb)	1,324.30	
Number of contigs	1,904	
Contig N50 length (Mb)	3.6	
Number of scaffolds	1,366	
Scaffold N50 length (Mb)	86.5	
Longest scaffold (Mb)	218.47	
Assembly metrics *		*Benchmark*
Consensus quality (QV)	59.2	*≥ 50*
*k*-mer completeness	100.0%	*≥ 95%*
BUSCO **	C:97.5%[S:97.2%,D:0.3%], F:0.5%,M:2.0%,n:8,338	*C ≥ 95%*
Percentage of assembly mapped to chromosomes	90.38%	*≥ 95%*
Sex chromosomes	Z	*localised homologous pairs*
Organelles	Mitochondrial genome: 16.75 kb	*complete single alleles*

* Assembly metric benchmarks are adapted from column VGP-2020 of “Table 1: Proposed standards and metrics for defining genome assembly quality” from Rhie *et al.* (2021). ** BUSCO scores based on the vertebrata_odb10 BUSCO set using version 5.4.3. C = complete [S = single copy, D = duplicated], F = fragmented, M = missing, n = number of orthologues in comparison. A full set of BUSCO scores is available at https://blobtoolkit.genomehubs.org/view/Larus_fuscus/dataset/GCA_963932225.1/busco.

**Table 3.** Chromosomal pseudomolecules in the genome assembly of *Larus fuscus*, bLarFus1.

INSDC accession	Name	Length (Mb)	GC%
OZ010753.1	1	218.47	41.5
OZ010754.1	2	167.92	41.0
OZ010755.1	3	128.68	41.5
OZ010756.1	4	94.81	42.5
OZ010758.1	5	83.13	41.0
OZ010759.1	6	73.36	43.0
OZ010760.1	7	58.1	43.5
OZ010761.1	8	55.73	44.0
OZ010762.1	9	49.4	44.0
OZ010763.1	10	25.19	44.5
OZ010764.1	11	22.05	45.5
OZ010765.1	12	17.98	47.0
OZ010766.1	13	17.47	46.5
OZ010767.1	14	14.96	48.0
OZ010768.1	15	13.69	49.0
OZ010769.1	16	10.09	48.5
OZ010770.1	17	9.36	50.5
OZ010771.1	18	8.79	52.5
OZ010772.1	19	8.7	51.5
OZ010773.1	20	7.91	53.0
OZ010774.1	21	6.75	54.5
OZ010775.1	22	6.57	50.5
OZ010776.1	23	4.26	58.0
OZ010777.1	24	3.3	59.0
OZ010778.1	25	1.07	59.0
OZ010779.1	26	0.84	63.0
OZ010780.1	27	0.51	65.0
OZ010781.1	28	0.48	62.0
OZ010782.1	29	0.42	56.5
OZ010783.1	30	0.29	67.0
OZ010784.1	31	0.17	61.0
OZ010757.1	Z	86.5	41.0
OZ010785.1	MT	0.02	45.0

The estimated Quality Value (QV) of the final assembly is 59.2 with *k*-mer completeness of 100.0%, and the assembly has a BUSCO v5.4.3 completeness of 97.5% (single = 97.2%, duplicated = 0.3%), using the vertebrata_odb10 reference set ( *n* = 8,338).

Metadata for specimens, BOLD barcode results, spectra estimates, sequencing runs, contaminants and pre-curation assembly statistics are given at https://links.tol.sanger.ac.uk/species/8915.

## Methods

### Sample acquisition and nucleic acid extraction

Several small samples of pectoral muscle were collected from a deceased lesser black-backed gull, *Larus fuscus*, specimen ID NHMUK014561644 (ToLID bLarFus1). This Laridae species was collected in Carmarthenshire, Wales, UK in 2021 as part of a disease surveillance programme carried out by WWT, in contribution to the Great Britain Wildlife Health Partnership, and stored at –20°C prior to sampling. Post-mortem examination by the WWT veterinarians revealed that the specimen was a female and that a shot pellet was the primary cause of death. The specimen was collected and identified by Rosa Lopez Colom (Wildfowl & Wetlands Trust).

The workflow for high molecular weight (HMW) DNA extraction at the Wellcome Sanger Institute (WSI) Tree of Life Core Laboratory includes a sequence of core procedures: sample preparation; sample homogenisation, DNA extraction, fragmentation, and clean-up. In sample preparation, the bLarFus1 sample was weighed and dissected on dry ice ( Jay *et al.*, 2023).

For sample homogenisation, muscle tissue was cryogenically disrupted using the Covaris cryoPREP ^®^ Automated Dry Pulverizer ( Narváez-Gómez *et al.*, 2023). HMW DNA was extracted using the Manual MagAttract v2 protocol ( Strickland *et al.*, 2023b). DNA was sheared into an average fragment size of 12–20 kb in a Megaruptor 3 system with speed setting 30 ( Todorovic *et al.*, 2023). Sheared DNA was purified by solid-phase reversible immobilisation ( Strickland *et al.*, 2023a): in brief, the method employs AMPure PB beads to eliminate shorter fragments and concentrate the DNA. The concentration of the sheared and purified DNA was assessed using a Nanodrop spectrophotometer and Qubit Fluorometer using the Qubit dsDNA High Sensitivity Assay kit. Fragment size distribution was evaluated by running the sample on the FemtoPulse system.

RNA was extracted from muscle tissue of bLarFus1 in the Tree of Life Laboratory at the WSI using the RNA Extraction: Automated MagMax™ *mir*Vana protocol ( do Amaral *et al.*, 2023). The RNA concentration was assessed using a Nanodrop spectrophotometer and a Qubit Fluorometer using the Qubit RNA Broad-Range Assay kit. Analysis of the integrity of the RNA was done using the Agilent RNA 6000 Pico Kit and Eukaryotic Total RNA assay.

Protocols developed by the WSI Tree of Life laboratory are publicly available on protocols.io ( Denton *et al.*, 2023).

### Sequencing

Pacific Biosciences HiFi circular consensus DNA sequencing libraries were constructed according to the manufacturers’ instructions. Poly(A) RNA-Seq libraries were constructed using the NEB Ultra II RNA Library Prep kit. DNA and RNA sequencing was performed by the Scientific Operations core at the WSI on Pacific Biosciences Sequel IIe (HiFi) and Illumina NovaSeq 6000 (RNA-Seq) instruments. Hi-C data were also generated from muscle tissue of bLarFus1 using the Arima-HiC v2 kit. The Hi-C sequencing was performed using paired-end sequencing with a read length of 150 bp on the Illumina NovaSeq 6000 instrument.

### Genome assembly, curation and evaluation

***Assembly***

HiFi reads were assembled using the ‘sanger-tol/genomeassembly’ pipeline ( Krasheninnikova *et al.*, 2024). Original assembly of HiFi reads is performed using Hifiasm ( Cheng *et al.*, 2021) with the --primary option. Haplotypic duplications were identified and removed with purge_dups ( Guan *et al.*, 2020). Hi-C reads are further mapped with bwa-mem2 ( Vasimuddin *et al.*, 2019) to the primary contigs, which are further scaffolded using the provided Hi-C data ( Rao *et al.*, 2014) in YaHS ( Zhou *et al.*, 2023) using the --break option. Scaffolded assemblies are evaluated using Gfastats ( Formenti *et al.*, 2022), BUSCO ( Manni *et al.*, 2021) and MERQURY.FK ( Rhie *et al.*, 2020).

The mitochondrial genome was assembled using MitoHiFi ( Uliano-Silva *et al.*, 2023), which runs MitoFinder ( Allio *et al.*, 2020) or MITOS ( Bernt *et al.*, 2013) and uses these annotations to select the final mitochondrial contig and to ensure the general quality of the sequence.

***Assembly curation***

The assembly was decontaminated using the Assembly Screen for Cobionts and Contaminants (ASCC) pipeline (article in preparation). Flat files and maps used in curation were generated in TreeVal ( Pointon *et al.*, 2023). Manual curation was primarily conducted using PretextView ( Harry, 2022), with additional insights provided by JBrowse2 ( Diesh *et al.*, 2023) and HiGlass ( Kerpedjiev *et al.*, 2018). Scaffolds were visually inspected and corrected as described by Howe *et al.* (2021). Any identified contamination, missed joins, and mis-joins were corrected, and duplicate sequences were tagged and removed. The sex chromosome was identified by synteny. The entire process is documented at https://gitlab.com/wtsi-grit/rapid-curation (article in preparation).

***Evaluation of the final assembly***

The final assembly was post-processed and evaluated with the three Nextflow ( Di Tommaso *et al.*, 2017) DSL2 pipelines “sanger-tol/readmapping” ( Surana *et al.*, 2023a), “sanger-tol/genomenote” ( Surana *et al.*, 2023b), and “sanger-tol/blobtoolkit” ( Muffato *et al.*, 2024). The pipeline sanger-tol/readmapping aligns the Hi-C reads with bwa-mem2 ( Vasimuddin *et al.*, 2019) and combines the alignment files with SAMtools ( Danecek *et al.*, 2021). The sanger-tol/genomenote pipeline transforms the Hi-C alignments into a contact map with BEDTools ( Quinlan & Hall, 2010) and the Cooler tool suite ( Abdennur & Mirny, 2020), which is then visualised with HiGlass ( Kerpedjiev *et al.*, 2018). It also provides statistics about the assembly with the NCBI datasets ( Sayers *et al.*, 2024) report, computes *k*-mer completeness and QV consensus quality values with FastK and MERQURY.FK, and a completeness assessment with BUSCO ( Manni *et al.*, 2021).

The sanger-tol/blobtoolkit pipeline is a Nextflow port of the previous Snakemake Blobtoolkit pipeline ( Challis *et al.*, 2020). It aligns the PacBio reads with SAMtools and minimap2 ( Li, 2018) and generates coverage tracks for regions of fixed size. In parallel, it queries the GoaT database ( Challis *et al.*, 2023) to identify all matching BUSCO lineages to run BUSCO ( Manni *et al.*, 2021). For the three domain-level BUSCO lineage, the pipeline aligns the BUSCO genes to the Uniprot Reference Proteomes database ( Bateman *et al.*, 2023) with DIAMOND ( Buchfink *et al.*, 2021) blastp. The genome is also split into chunks according to the density of the BUSCO genes from the closest taxonomically lineage, and each chunk is aligned to the Uniprot Reference Proteomes database with DIAMOND blastx. Genome sequences that have no hit are then chunked with seqtk and aligned to the NT database with blastn ( Altschul *et al.*, 1990). All those outputs are combined with the blobtools suite into a blobdir for visualisation.

The genome assembly and evaluation pipelines were developed using the nf-core tooling ( Ewels *et al.*, 2020), use MultiQC ( Ewels *et al.*, 2016), and make extensive use of the Conda package manager, the Bioconda initiative ( Grüning *et al.*, 2018), the Biocontainers infrastructure ( da Veiga Leprevost *et al.*, 2017), and the Docker ( Merkel, 2014) and Singularity ( Kurtzer *et al.*, 2017) containerisation solutions.

Table 4 contains a list of relevant software tool versions and sources.

**Table 4.** Software tools: versions and sources.

Software tool	Version	Source
BEDTools	2.30.0	https://github.com/arq5x/bedtools2
BLAST	2.14.0	ftp://ftp.ncbi.nlm.nih.gov/blast/executables/blast+/
BlobToolKit	4.3.7	https://github.com/blobtoolkit/blobtoolkit
BUSCO	5.4.3 and 5.5.0	https://gitlab.com/ezlab/busco
bwa-mem2	2.2.1	https://github.com/bwa-mem2/bwa-mem2
Cooler	0.8.11	https://github.com/open2c/cooler
DIAMOND	2.1.8	https://github.com/bbuchfink/diamond
fasta_windows	0.2.4	https://github.com/tolkit/fasta_windows
FastK	427104ea91c78c3b8b8b49f1a7d6bbeaa869ba1c	https://github.com/thegenemyers/FASTK
Gfastats	1.3.6	https://github.com/vgl-hub/gfastats
GoaT CLI	0.2.5	https://github.com/genomehubs/goat-cli
Hifiasm	0.19.8-r603	https://github.com/chhylp123/hifiasm
HiGlass	44086069ee7d4d3f6f3f0012569789ec138f42b84 aa44357826c0b6753eb28de	https://github.com/higlass/higlass
Merqury.FK	d00d98157618f4e8d1a9190026b19b471055b22e	https://github.com/thegenemyers/MERQURY.FK
MitoHiFi	3	https://github.com/marcelauliano/MitoHiFi
MultiQC	1.14, 1.17, and 1.18	https://github.com/MultiQC/MultiQC
NCBI Datasets	15.12.0	https://github.com/ncbi/datasets
Nextflow	23.04.0-5857	https://github.com/nextflow-io/nextflow
PretextView	0.2	https://github.com/sanger-tol/PretextView
purge_dups	1.2.5	https://github.com/dfguan/purge_dups
samtools	1.16.1, 1.17, and 1.18	https://github.com/samtools/samtools
sanger-tol/ascc	-	https://github.com/sanger-tol/ascc
sanger-tol/ genomeassembly	0.10.0	https://github.com/sanger-tol/genomeassembly
sanger-tol/ genomenote	1.1.1	https://github.com/sanger-tol/genomenote
sanger-tol/ readmapping	1.2.1	https://github.com/sanger-tol/readmapping
Seqtk	1.3	https://github.com/lh3/seqtk
Singularity	3.9.0	https://github.com/sylabs/singularity
TreeVal	1.0.0	https://github.com/sanger-tol/treeval
YaHS	1.2a.2	https://github.com/c-zhou/yahs

### Wellcome Sanger Institute – Legal and Governance

The materials that have contributed to this genome note have been supplied by a Darwin Tree of Life Partner. The submission of materials by a Darwin Tree of Life Partner is subject to the **‘Darwin Tree of Life Project Sampling Code of Practice’**, which can be found in full on the Darwin Tree of Life website here. By agreeing with and signing up to the Sampling Code of Practice, the Darwin Tree of Life Partner agrees they will meet the legal and ethical requirements and standards set out within this document in respect of all samples acquired for, and supplied to, the Darwin Tree of Life Project.

Further, the Wellcome Sanger Institute employs a process whereby due diligence is carried out proportionate to the nature of the materials themselves, and the circumstances under which they have been/are to be collected and provided for use. The purpose of this is to address and mitigate any potential legal and/or ethical implications of receipt and use of the materials as part of the research project, and to ensure that in doing so we align with best practice wherever possible. The overarching areas of consideration are:

•     Ethical review of provenance and sourcing of the material

•     Legality of collection, transfer and use (national and international) 

Each transfer of samples is further undertaken according to a Research Collaboration Agreement or Material Transfer Agreement entered into by the Darwin Tree of Life Partner, Genome Research Limited (operating as the Wellcome Sanger Institute), and in some circumstances other Darwin Tree of Life collaborators.

## Data Availability

European Nucleotide Archive: *Larus fuscus* (lesser black-backed gull). Accession number PRJEB71570; https://identifiers.org/ena.embl/PRJEB71570 ( Wellcome Sanger Institute, 2024). The genome sequence is released openly for reuse. The *Larus fuscus* genome sequencing initiative is part of the Darwin Tree of Life (DToL) project. All raw sequence data and the assembly have been deposited in INSDC databases. The genome will be annotated using available RNA-Seq data and presented through the Ensembl pipeline at the European Bioinformatics Institute. Raw data and assembly accession identifiers are reported in Table 1 and Table 2.

## Author information

Members of the Darwin Tree of Life Barcoding collective are listed here: https://doi.org/10.5281/zenodo.4893703.

Members of the Wellcome Sanger Institute Tree of Life Management, Samples and Laboratory team are listed here: https://doi.org/10.5281/zenodo.10066175.

Members of Wellcome Sanger Institute Scientific Operations: Sequencing Operations are listed here: https://doi.org/10.5281/zenodo.10043364.

Members of the Wellcome Sanger Institute Tree of Life Core Informatics team are listed here: https://doi.org/10.5281/zenodo.10066637.

Members of the Tree of Life Core Informatics collective are listed here: https://doi.org/10.5281/zenodo.5013541.

Members of the Darwin Tree of Life Consortium are listed here: https://doi.org/10.5281/zenodo.4783558.
