# Supplementary material for: The genome sequence of the lesser black-backed gull, Larus fuscus Linnaeus, 1758
Source: Wellcome Open Res. 2024 Oct 15;9:444. Originally published 2024 Aug 12. [Version 2] doi: 10.12688/wellcomeopenres.22766.2 (PMC12603531; doi:10.12688/wellcomeopenres.22766.2)
Supplement: Peer review history

# Reviewer report: Reviewer response for version 2

**DOI:** 10.21956/wellcomeopenres.25605.r135894

**Published:** 2025-11-11

**Authors:** Rachael Herman

**Affiliations:** 1 Stony Brook University, Stony Brook, New York, USA; 2 Columbia University Department of Ecology Evolution and Environmental Biology (Ringgold ID: 189435), New York, New York, USA

The paper presents a chromosome-level genome assembly for the lesser black-backed gull, *Larus fuscus* (LBBG), as part of the Darwin Tree of Life Project. The individual sequenced was an adult female specimen collected in Carmarthenshire, Wales, UK, in 2021.The assembly was generated using a combination of Pacific HiFi long reads and Hi-C chromosome conformation data for scaffolding. The total length of the assembly is 1,324.30 Mb, and ~90% was successfully scaffolded into 32 chromosomal pseudomolecules, representing 31 autosomes and the Z sex chromosome. 

 While the authors provide some basic background on LBBG, I think this section would benefit from covering any previous genetics research and/or genome assemblies. How will this new reference genome improve upon future research on the LBBG?

Are sufficient details of methods and materials provided to allow replication by others?

Yes

Is the rationale for creating the dataset(s) clearly described?

Partly

Are the datasets clearly presented in a useable and accessible format?

Yes

Are the protocols appropriate and is the work technically sound?

Yes

Reviewer Expertise:

population genetics, population genomics, epigenetics, population ecology

I confirm that I have read this submission and believe that I have an appropriate level of expertise to confirm that it is of an acceptable scientific standard.

# Reviewer report: Reviewer response for version 2

**DOI:** 10.21956/wellcomeopenres.25605.r105086

**Published:** 2024-10-28

**Authors:** Yang Liu

**Affiliations:** 1 Sun Yat-sen University, Guangzhou, China

I'm satisfied with the revision.

Are sufficient details of methods and materials provided to allow replication by others?

Partly

Is the rationale for creating the dataset(s) clearly described?

Yes

Are the datasets clearly presented in a useable and accessible format?

Yes

Are the protocols appropriate and is the work technically sound?

Yes

Reviewer Expertise:

Evolutionary biology, Speciation. conservation genomics

I confirm that I have read this submission and believe that I have an appropriate level of expertise to confirm that it is of an acceptable scientific standard.

# Reviewer report: Reviewer response for version 2

**DOI:** 10.21956/wellcomeopenres.25605.r105087

**Published:** 2024-10-22

**Authors:** Marcelo de Bello AbdilzadehCioffi, Fernando Souza, Francisco de Menezes Cavalcante Sassi

**Affiliations:** 1 Universidade Fedral de São Carlos, São Carlos, Brazil; 2 Universidade Federal de São Carlos, São Paulo, Brazil

The authors indicate that the bird's sex was ascertained by post-mortem investigation and provided details regarding the sample collecting site. Nonetheless, some issues highlighted in the initial revision remain unresolved (e.g., the inconsistency between the 2n values identified herein and those from prior research must be addressed and analyzed / It remains unclear why this specific Larus species was chosen, as the discovery of deceased individuals should not serve as a legitimate rationale for selection).

Are sufficient details of methods and materials provided to allow replication by others?

No

Is the rationale for creating the dataset(s) clearly described?

Partly

Are the datasets clearly presented in a useable and accessible format?

Yes

Are the protocols appropriate and is the work technically sound?

Yes

Reviewer Expertise:

Animal Genomics

We confirm that we have read this submission and believe that we have an appropriate level of expertise to confirm that it is of an acceptable scientific standard, however we have significant reservations, as outlined above.

# Reviewer report: Reviewer response for version 1

**DOI:** 10.21956/wellcomeopenres.25070.r98763

**Published:** 2024-10-09

**Authors:** Yang Liu

**Affiliations:** 1 Sun Yat-sen University, Guangzhou, China

This manuscript presents a chromosome-level genome assembly for *Larus fuscus*. The authors provide a concise overview of the background of this species. The necessity to undertake this work was twofold: 1) Resources for speciation genomics; genomic analysis elucidates the extent and impact of hybridization and facilitates the understanding of genetic exchange between species. 2) Conservation genomics: although this gull is categorized as 'Least Concern' on the IUCN Red List, its conservation status in the UK is amber due to a substantial population decline. 

 Both the manuscript is well written and the genome assembly was executed properly. I just have a few minor comments, mostly regarding transparency and clarity.

 1. In the background section, the authors mention that the lesser black-backed gull consists of several subspecies. However, they did not indicate which subspecies the sequencing sample belonged to. If the authors can identify this bird at the subspecies level and provide more specific information, it would be beneficial for other researchers to use this genomic data.

 2. In the first paragraph of the genome sequence report, the authors state that a total of 18.14 Gb raw data provides 30-fold coverage for *L. fuscus* genome. However, the size of the bird genome is usually around 1.3 Gb. It is not possible to achieve 30-fold coverage with 18 Gb raw data. Additionally, why did the authors construct three PacBio Sequel libraries as shown in Table 1?

 3. In the second paragraph of the genome sequence report, the authors write: “The final assembly has a total length of 1,324.30 Mb in 1,366 sequence scaffolds with a scaffold N50 of 86.5 Mb (Table 2), and 537 gaps. The total count of gaps in the scaffolds is 537.” The number of gaps in the scaffolds is mentioned twice. I suggest revising these sentences for clarity.

 4. Why did the authors use different BUSCO datasets for comparison in Table 1 (vertebrata_odb10) and Figure 2 (aves_ odb10) instead of being consistent?

 .

Are sufficient details of methods and materials provided to allow replication by others?

Partly

Is the rationale for creating the dataset(s) clearly described?

Yes

Are the datasets clearly presented in a useable and accessible format?

Yes

Are the protocols appropriate and is the work technically sound?

Yes

Reviewer Expertise:

Evolutionary biology, Speciation. conservation genomics

I confirm that I have read this submission and believe that I have an appropriate level of expertise to confirm that it is of an acceptable scientific standard.

# Reviewer report: Reviewer response for version 1

**DOI:** 10.21956/wellcomeopenres.25070.r98758

**Published:** 2024-09-18

**Authors:** Marcelo de Bello AbdilzadehCioffi, Fernando Souza, Francisco de Menezes Cavalcante Sassi

**Affiliations:** 1 Universidade Fedral de São Carlos, São Carlos, Brazil; 2 Universidade Federal de São Carlos, São Paulo, Brazil

**General Comments**

 This work presents a genome assembly from a female individual of *Larus fuscus* (the lesser black-backed gull; Chordata; Aves; Charadriiformes; Laridae) through a combination of updated methods currently used for performing a genome assembly at the chromosomal level (i.e., a combination of PacBio Hifi and Hi-C sequencing data). Besides the issues highlighted below, I also call attention to one specific problem with the current results. The authors state that the assembly sequence was assigned to 32 chromosomal-level scaffolds, representing 31 autosomes and the Z sex chromosome. However, previous data conducted on this species [(Hammar, Hereditas 65: 29-58 (1970)], as well as in the congeneric species *Larus argentus* (Hansmann et al., 2009; doi: 10.1159/000251965) clearly display a 2n=70 for these species. Discrepancies in the findings arise from the fact that almost all genome descriptions released completely ignore (both novel and/or previous) chromosomal data of the investigated species.  Almost none of the genomes being currently produced present such a connection to their true chromosomes. A DNA scaffold—regardless of its size or completeness—can never be fully regarded as a chromosome and, regrettably, direct information linked with the target species karyotype is not usually (in fact, rarely) provided. In conclusion, without a karyotypic study related to the individual who was examined, at least this discrepancy between the values ​​found here and previous studies must be considered and discussed.

**Data Note**

- **Is the rationale for creating the dataset(s) clearly described?**

The motivation for the construction of the datasets is well articulated, however, some aspects lack sufficient clarity. It is not fully clear why this particular Larus species was selected. Besides, did the authors attempt to ascertain the sequenced individual's subspecies classification? If not, what is the reason? Furthermore, some additional information is missing, as described in the third item of this evaluation. 

- **Are the protocols appropriate and is the work technically sound?**

Yes, the authors used one of the most updated methods currently used for performing a genome assembly at the chromosomal level (i.e., a combination of PacBio Hifi and Hi-C sequencing data). The authors indicated every protocol used in genome assembly, as well as the software versions and sources, in a well-organized table (Table 4). However, ideally, 3-4 transcriptomes from different tissue types could have been performed and used for more assertive annotation.

- **Are sufficient details of methods and materials provided to allow replication by others?**

Negative. The genome assembly procedures are freely accessible; yet, the work lacks critical information. The species identification in the manuscript is insufficiently described. The authors mention: “This Laridae species was collected in Carmarthenshire in 2021 as part of a disease surveillance program carried out by WWT, in contribution to the Great Britain Wildlife Health Partnership, and stored at -20°C before sampling.” Nonetheless, references to city names and acronyms must be clarified upon their first occurrence in the text. The location of "Carmarthenshire" is ambiguous; it is unclear which nation it belongs to and if it is a city, town, or village. Geographical coordinates would be also helpful for clarification. Besides problems with acronyms that are seen throughout the whole text, the material and methods section lacks important information regarding the individual being sequenced. The authors fail to provide the method used to identify this species and its sex, both of which are crucial for the replicability of the findings. Larus is a genus recognized for its complex taxonomy, including subspecies, as stated in the introductory section. The lack of information on how they determined the individual's sex is significant, considering members of the species are monomorphic, exhibiting little or no morphological change between sexes. Besides, the details of the material sample are ambiguous. They discovered a deceased animal with a gunshot wound and used this specimen for sequencing. This information seems to lack enough details on procedures and materials necessary for replication by others. While the authors include information on a larger surveillance effort, the current study fails to clarify why they selected a deceased animal instead of collecting fresh samples, particularly given that the species is neither endangered nor rare in nature. Regarding authorizations to conduct the study, the authors mention that “Each transfer of samples is further undertaken according to a Research Collaboration Agreement or Material Transfer Agreement entered into by the Darwin Tree of Life Partner, Genome Research Limited (operating as the Wellcome Sanger Institute), and in some circumstances other Darwin Tree of Life collaborators.” This statement lacks specificity and does not include any authorization number or verifiable information. For the study to be replicable, the data must be readily accessible. 

- **Are the datasets clearly presented in a usable and accessible format?**

The authors provide access to the Embl project, where the genome assembly is accessible for chromosome-by-chromosome access. The authors give access to both the raw sequencing data and the RNAseq data used for genome annotation. All data is now accessible for download in several gold-standard formats.

Are sufficient details of methods and materials provided to allow replication by others?

No

Is the rationale for creating the dataset(s) clearly described?

Partly

Are the datasets clearly presented in a useable and accessible format?

Yes

Are the protocols appropriate and is the work technically sound?

Yes

Reviewer Expertise:

Animal Genomics

We confirm that we have read this submission and believe that we have an appropriate level of expertise to confirm that it is of an acceptable scientific standard, however we have significant reservations, as outlined above.

## References

[1] : The karyotypes of thirty-one birds. *Hereditas* .2009;65(1) : 10.1111/j.1601-5223.1970.tb02306.x 29-58 10.1111/j.1601-5223.1970.tb02306.x

[2] : Cross-species chromosome painting corroborates microchromosome fusion during karyotype evolution of birds. *Cytogenet Genome Res* .2009;126(3) : 10.1159/000251965 281-304 10.1159/000251965 20068299
